# Sex Differences Exist in the Relationship Between Vertical Jump Performance Biomechanical Parameters and Hamstring/Quadriceps Ratio in Adolescent U18 Track and Field Athletes

**DOI:** 10.3390/sports12110295

**Published:** 2024-10-28

**Authors:** Vassilios Panoutsakopoulos, Mariana C. Kotzamanidou, Vasiliki Manou

**Affiliations:** 1 School of Physical Education and Sport Science at Thessaloniki, Aristotle University of Thessaloniki, 54124 Thessaloniki, Greece; 2 Faculty of Health Sciences, Metropolitan College of Thessaloniki, 54624 Thessaloniki, Greece; 3 Department of Coaching and Physical Education, School of Sport Sciences and Physical Education, Metropolitan College of Thessaloniki, 54624 Thessaloniki, Greece; 4 Institute of Occupational Science & Rehabilitation, Metropolitan College, 15125 Athens, Greece

**Keywords:** track and field, adolescence, vertical jumps, isokinetic dynamometry, biomechanical analysis, hamstring to quadriceps ratio, conventional ratio, sex differences, inter-limb asymmetry

## Abstract

Isokinetic metrics are suggested to be related to vertical jump performance, but little is known about the possible sex differences in this relationship in adolescent track and field athletes. The aim of the study was to examine the possible sex differences in the strength of the relationship between the kinetic parameters of the vertical squat jump with arms akimbo, the countermovement jump with arms akimbo, and the countermovement jump with free arm swing with the isokinetic parameters of the knee extensor and flexor muscles (angular velocities: 60°/s and 300°/s). In total, 35 (males: 21, females: 14) U18 track and field athletes were examined. The results revealed that the Men U18 group had higher vertical jump performance, higher values in the kinetic parameters of the vertical jump tests, higher knee extensor torque, and higher knee flexor torque in the non-dominant leg compared to the Women U18 group. Significant (*p* < 0.05) sex differences were observed in the relationship between the isokinetic parameters and the vertical jump performance metrics, as the Women U18 athletes relied more on the knee flexor torque than Men U18 athletes, and demonstrated a stronger negative relationship between selected isokinetic metrics and the pre-stretch gain in the vertical jump tests. In conclusion, it is recommended that young female track and field athletes minimize interlimb asymmetry and improve the convention ratio in their non-dominant leg to optimize vertical jump performance.

## 1. Introduction

The physical fitness status of athletes is commonly assessed using vertical jump tests, where various aspects of the strength and conditioning capabilities are evaluated [1]. For example, the vertical squat jump test (SJ) is considered to assess the concentric muscular strength application capability of the lower extremity extensor muscles [2]. In addition, the vertical countermovement jump test (CMJ) is widely used to monitor the performance gain (namely, the increase in jump height) resulting from the pre-stretch occurring during the downward phase of the CMJ [3]. Another common vertical jump test is the CMJ with the use of an arm swing. With this vertical jump test, the intra-segmental neuromuscular coordination across the upper and lower limbs and the resulting energy flow of the work generated at the shoulder joint to the ground via the proximal-to-distal sequencing of the lower limb action is depicted by the gain in performance from the CMJ with no arm swing to the CMJ with the arm swing [4,5,6].

The importance of jumping performance in adolescent track and field athletes is highlighted in the respective literature [7]. Previous research has shown that track and field athletes outperform athletes from other sports, a finding that is attributed to the larger power outputs [8,9,10,11], as past research has shown that track and field athletes execute the vertical jump tests relying on a force-dominant pattern that distinguishes them from athletes with other sport backgrounds [8,10,12]. This larger power output in track and field athletes is related to both the demands of a powerful execution of the technique in the vast majority of the track and field disciplines and the sport-specific execution of the jumping exercises that is a characteristic in athletics training [13,14,15]. Nevertheless, performance in the vertical jump tests is not homogeneous among track and field athletes, as differences have been observed due to the discipline [11,16,17], the level of performance [18,19], the part of the training period within the season [20,21,22], sex [23,24] and age within adolescence [25]. The sex difference between male and female track and field athletes is enlarged during the progression of adolescence, since male athletes grow more than females in terms of body size, muscle mass, strength and power [26]. Despite the changes in vertical jump performance during adolescence, post-pubertal adolescent track and field athletes execute the vertical squat jump in a similar force-dependent pattern to young adult track and field athletes [27].

Besides the evaluation of the physical condition in track and field athletes, the results of the kinetic parameters of the vertical jumps, i.e., the force and power outputs, the rate of force development, the temporal structure, etc., are of importance regarding the examination of the possible prediction of performance in track and field events such as the 100 m sprint, the long jump, etc. [19,28,29,30,31,32]. The same investigation was conducted using the outcomes of isokinetic dynamometry, namely peak torque of the lower limb extensors and flexors [33,34,35]. Similarly to the vertical jumps, differences in the isokinetic parameters among track and field athletes were found due to discipline [36,37,38], sex [38,39] and age within adolescence [36]. The isokinetic testing in both slow (i.e., 60°/s) and fast (i.e., 300°/s) angular velocities [40] provides valuable information for performance, training effects and injury prevention in track and field athletes. Nevertheless, the information that is attracting the focus of coaches and researchers is the conventional hamstring to quadriceps ratio (H/Q) ratio and the interlimb asymmetry regarding the isokinetic parameters [41]. Interlimb asymmetry in isokinetic metrics was found in adolescent track and field athletes and is assumed to be related to injury risk [36]. In addition, sex differences were found in the isokinetic leg press test, with males been stronger that females [42].

Past research has studied if isokinetic metrics are related to jumping performance [43]. Evidence suggests that the H/Q ratio is related to jumping ability in a variety of athletes with different sports, such as soccer players [44], volleyball players [45], sailors [46], and rugby players [47], among others. Regarding the vertical jump kinetic parameters specifically, the H/Q ratio was found to be related to the mechanical power of the CMJ [44,48]. Even though an association is suggested to exist between the vertical jump and isokinetic metrics in young athletes [49], no such information exists for international level adolescent track and field athletes and for the sex differences in this relationship, especially in terms of which isokinetic parameter each sex relies on more to achieve the optimum vertical jump height.

The purpose of this study was to examine the possible sex differences in the strength of the relationship between the kinetic parameters of vertical jump tests and the isokinetic parameters of the knee extensor and knee flexor muscles. The following hypotheses were made:

1. The knee extensor torque will not be different between the lower limbs;
2. The knee extensor torque will correlate with vertical jump performance and kinetics;
3. No sex differences will be present regarding the strength of the relationship between male and female adolescent track and field athletes that have competed in international athletics events.

## 2. Materials and Methods

### 2.1. Design of the Study

To examine the hypothesis of the study, a cross-sectional study was applied, where the participants performed the assessment of the isokinetic torque of the knee extensors and flexors, and the vertical jump tests in random order.

### 2.2. Participants

A total of 35 post-pubertal track and field athletes were tested. Of this convenience sample, 21 competed for the Men’s U18 category (17.3 ± 0.9 yrs, 1.80 ± 0.07 m, 81.0 ± 21.4 kg, 23.1 ± 2.2 kg/m^2^; 8 sprinters, 6 jumpers, and 7 throwers) and 14 competed for the Women’s U18 category (17.3 ± 1.0 yrs, 1.70 ± 0.06 m, 66.4 ± 17.4 kg, 21.7 ± 3.6 kg/m^2^; 5 sprinters, 4 jumpers, and 5 throwers).

The inclusion criteria were that the athletes systematically participated in their training program (12–15 h/wk) and the selection to compete for the National U18 Track and Field team. The exclusion criterion was the experience of an injury or illness that prohibited training for more than 3 days for a period of 6 months before testing.

The tests were conducted in the middle of the outdoor competitive season, after a rest day following the last intense training session. The experimental procedure was in accordance with the Declaration of Helsinki and the Research Ethics Code of the Aristotle University of Thessaloniki (ethical approval granted by the Research Committee of the Aristotle University of Thessaloniki with the protocol code 7233/09.11.97).

### 2.3. Procedure

Barefoot body height and mass were assessed using the Delmac PS400L scale (Delmac Instruments S.A., Athens, Greece) and the Seca 220 stadiometer (Seca Deutschland, Hamburg, Germany). The dominant lower limb was defined as the preferred side to perform a running long jump task [32].

Warm-up was conducted by running for 10 min on a motorized Technogym treadmill (Technogym S.p.A., Cesena, Italy). A session of 6 min dynamic stretching exercises followed, where the range of motion and the intensity of movement gradually increased.

After the warm-up, participants randomly performed the isokinetic or the vertical jump tests, with a 15 min interval between testing sessions. No familiarization sessions were conducted as the participants were familiar with the testing procedures, since the tests were part of their regular physical fitness screening by the National Amateur Athletics Federation.

#### 2.3.1. Execution of the Vertical Jump Tests

The AMTI OR6-5-1 force-plate (AMTI, Newton, MA, USA) was used for the assessment of the performance (jump height), as well as the spatiotemporal and kinetic parameters of the tested vertical jumps. The sampling and the cut-off frequency were set at 1 kHz and 20 Hz, respectively.

The participants performed, in a random order, three vertical jump tests: the squat jump with the arms kept akimbo (SJ-A), the countermovement jump with the arms kept akimbo (CMJ-A), and the countermovement jump with free arm swing (CMJ-F). In all jumping tests, the instruction was to “jump as high and as fast as you can”. Within the same vertical jump tests, a 60 s interval was allowed, whereas a rest of 3 min was provided between different vertical jump tests. The procedure to execute the above-mentioned vertical jump tests was conducted following the methods described in detail elsewhere [10,50]. No specific instruction was provided concerning the knee flexion during the countermovement [51].

#### 2.3.2. Execution of the Isokinetic Tests

The Cybex Norm isokinetic dynamometer (CYBEX Division of Lumex, Ronkonkoma, NY, USA) was used for the measurement of the isokinetic torque during the concentric contractions of the knee extensor and flexor muscles. Participants were seated and stabilized on the dynamometer’s chair with tight velcro straps at the upper thigh, waist, and trunk. The inclination of the back of the chair was adjusted so that the hip angle was at 115°. The chair was also adjusted so that the axis of rotation of the dynamometer was aligned with the most prominent point of the medial femoral epicondyle. Afterwards, the correction for gravity was estimated, following the definition of the full knee joint extension. For the isokinetic tests, the range of motion for the knee joint was fixed to the range of 0° (=full knee extension) to 90° knee flexion for all participants.

The isokinetic tests were executed in a random order. Three maximal concentric knee extension and flexion attempts at the selected angular velocities of 60°/s and 300°/s [38,40] were performed in a randomized order referring to the dominant leg. The inter-test rest was 3 min. Visual feedback by means of displaying torque values on a screen and constant encouragement of the participants to outperform their previous trial in both knee extension and flexion was provided.

### 2.4. Data Analysis

#### 2.4.1. Analysis of the Vertical Jump Tests

The recording and the analysis of the vertical ground reaction forces (vGRF) were carried out with the modules of the K-Dynami 2018 software (©: Iraklis A. Kollias, Biomechanics Laboratory, Aristotle University of Thessaloniki, Thessaloniki, Greece). The recorded vGRF data were smoothed using a 2nd-order digital low pass Butterworth recursive filter. Using the trapezoid rule, the first-time integral of the net vGRF was used to calculate the body center of mass (BCM) vertical take-off velocity and consequently the jump height. Based on the classical equations of motion, the recorded vGRF time history, and the participants’ body mass, and using methods described in detail elsewhere [10,52], the spatiotemporal parameters, namely the total impulse time, the lowering of the BCM, the total vertical BCM displacement, the time to achieve peak vGRF and peak power, as well as the kinetic parameters (relative peak vGRF, rate of force development, peak relative power), were calculated. The pre-stretch gain was extracted as the percentage chance of the jump height in CMJ-A compared to SJ-A, whereas the arm swing augmentation ratio (ASAR) was calculated as the percentage chance of the jump height in CMJ-F compared to CMJ-A. For all vertical jump tests, only the trial with the highest jump height was included in the analysis.

#### 2.4.2. Analysis of the Isokinetic Tests

During both isokinetic tests (60°/s and 300°/s), only the attempt with the highest recorded peak torque, as well as the angle at which it was achieved, for both knee extensors and flexors, was selected for further analysis. To conduct the comparisons, the relative to body mass peak torque was selected. The conventional H/Q ratio was computed as the concentric flexor/extensor ratio [53]. The inter-limb difference was expressed as the absolute value of the percentage difference between the dominant and non-dominant leg. The examination of the inter-limb asymmetry in the isokinetic parameters was carried out using the symmetry angle [54], which was calculated using Equation (1).

(1) $$symmetry\;angle=\frac{\left(45^{ο}-\arctan\left(\frac{\mathrm{dominant}\;\mathrm{leg}}{\mathrm{non-dominant}\;\mathrm{leg}}\right)\right)}{90^{ο}}\times 100\%$$

In the case that the condition depicted in Equation (2) is true:

(2) $$\left(45^{ο}-\arctan\left(\frac{\mathrm{dominant}\;\mathrm{leg}}{\mathrm{non-dominant}\;\mathrm{leg}}\right)\right)>90^{o}$$

then Equation (1) was replaced by Equation (3), as shown below:

(3) $$symmetry\;angle=\frac{\left(45^{ο}-\arctan\left(\frac{\mathrm{dominant}\;\mathrm{leg}}{\mathrm{non-dominant}\;\mathrm{leg}}\right)-180^{ο}\right)}{90^{ο}}\times 100\%$$

The positive symmetry angle implied that the direction of asymmetry was towards the dominant side, whereas a negative symmetry angle indicated a larger dominant-side value. Nevertheless, the absolute symmetry angle values were utilized in the analysis to examine the magnitude of asymmetry [55].

### 2.5. Statistical Analysis

Based on the calculations for the a priori estimation of the sample size using the G*power v.3.1.9.7 software [56,57], the examined sample of 35 athletes corresponded to 0.6 power for a 0.8 effect size at *a* = 0.05 for the between-sex comparisons.

The normality of distribution of the data was checked using the Shapiro–Wilk test (*p* > 0.05). The equality of variance was examined with Levene’s test (*p* > 0.05). Based on the results of these tests, parametric statistical tests were applied.

Possible sex differences were examined using the independent samples *t*-test. The effect size of the comparison was interpreted using Hedges’ *g*, where values of *g* ≥ 0.2, *g* ≥ 0.5, and *g* ≥ 0.8, represented small, medium, and large effect sizes, respectively.

The possible linear relationship between performance in the vertical jump tests and the examined isokinetic parameters was checked with the Pearson correlation coefficient (*r*) separately for the Men U18 and the Women U18 groups. Values of *r* within the range of 0.00–0.09, 0.10–0.39, 0.40–0.69, 0.70–0.89 and 0.90–1.00 were interpreted as negligible, weak, moderate, strong and very strong relationships, respectively [58]. Significant correlations were followed-up for the comparison of *r* between Men U18 and the Women U18 by implementing Fisher’s r-to-Z transformation using the Psychometrica freeware calculator [59].

The level of significance for all analyses was set at a = 0.05. The IBM SPSS Statistics v.29.0.1.0 software (ΙΒΜ Corp., Armonk, NY, USA) was used to run the statistical tests.

## 3. Results

### 3.1. Results for the Vertical Jump Tests

#### 3.1.1. Squat Jump with Arms Akimbo

The results for the SJ-A are presented in Table 1. Significant (*p* < 0.05) sex differences (large effect size) were revealed for jump height, relative vGRF, rate of force development, relative power, and vertical BCM displacement, which were larger in Men U18. Women U18 demonstrated a significantly (*p* < 0.05) higher time to achieve peak vGRF. Jump height was significantly (*p* < 0.05) strongly correlated in both groups with relative power, with vertical BCM displacement in the Men U18 group (moderate correlation), and with relative vGRF in the Women U18 group (strong correlation). The relationship between jump height and relative vGRF was significantly (*p* < 0.05) greater in the Women U18 compared to the Men U18 group.

#### 3.1.2. Countermovement Jump with Arms Akimbo

The results for the CMJ-A are presented in Table 2. Significant (*p* < 0.05) sex differences (large effect size) were revealed for jump height, relative vGRF, rate of force development, relative power, BCM lowering and vertical BCM displacement, which were larger in Men U18. Jump height was significantly (*p* < 0.05) strongly correlated in both groups with relative power, without a significant (*p* > 0.05) difference in the magnitude of the relationship between Men U18 and Women U18.

#### 3.1.3. Countermovement Jump with Free Arm Swing

The results for the CMJ-F are presented in Table 3. Significant (*p* < 0.05) sex differences (large effect size) were revealed for jump height, relative vGRF, relative power, and vertical BCM displacement, which were larger in Men U18. Jump height was significantly (*p* < 0.05) correlated with relative power (strong correlation in Men U18 and moderate correlation in Women U18) and with relative vGRF in the Men U18 group (moderate correlation). The relationship between jump height and relative power was not significantly (*p* > 0.05) different between Men U18 and Women U18.

### 3.2. Results for the Isokinetic Tests

#### 3.2.1. Isokinetic Concentric Contraction—60°/s

The results for the isokinetic tests at the angular velocity of 60°/s are presented in Table 4. Significant (*p* < 0.05) sex differences (large effect size) were revealed for the relative torque of the knee extensors in both the dominant and non-dominant leg, as well as for the relative torque of the knee flexors in the dominant leg. In these parameters, Men U18 had larger values than Women U18.

In the Men U18 group, the only significant (*p* < 0.05) correlation was revealed between the jump height of the SJ-A and the angle to achieve peak knee flexor torque of the non-dominant leg (Table 5). As in the Women U18 group, the knee extensor relative torque of the dominant leg was significantly (*p* < 0.05) correlated with the jump height of the SJ-A and the CMJ-F, while the knee extensor relative torque of the non-dominant leg was significantly (*p* < 0.05) correlated with the jump height of CMJ-F.

In addition, the relative torque of knee flexors in both the dominant and non-dominant leg was significantly (*p* < 0.05) correlated with the jump height in all vertical jump tests, as well as with the pre-stretch gain. Finally, the conventional H/Q ratio for the dominant leg and the symmetry angle of the inter-limb comparison for the angle to achieve the peak torque for the knee flexors was significantly (*p* < 0.05) correlated with the jump height of CMJ-A.

The relationship between the relative torque of knee flexors in both the dominant and non-dominant leg with the jump height of the vertical jump tests and the pre-stretch gain was significantly (*p* < 0.05) greater in the Women U18 group than the Men U18 group. The comparison of the correlation coefficients between the angle to achieve peak knee flexor torque in the non-dominant leg and the conventional H/Q ratio for the dominant leg with SJ-A performance, as well as between the symmetry angle of the inter-limb comparison for the angle to achieve the peak torque for the knee flexors with the jump height of CMJ-A, revealed significant (*p* < 0.05) sex differences.

#### 3.2.2. Isokinetic Concentric Contraction—300°/s

The results for the isokinetic tests at the angular velocity of 300°/s are presented in Table 6. Similar to the respective results for the isokinetic tests at the angular velocity of 60°/s, significant (*p* < 0.05) sex differences (large effect size) were revealed for the relative torque of the knee extensors in both the dominant and non-dominant leg, as well as for the relative torque of the knee flexors in the dominant leg.

In addition, the inter-limb symmetry angles for the relative torque of the knee flexors were significantly (*p* < 0.05) different between groups. For all the parameters where significant sex differences were observed, Men U18 had larger values than Women U18.

In the Women U18 group, the pre-stretch gain was significantly (*p* < 0.05) correlated with the dominant leg’s knee flexors and extensor relative torque, the relative torque of the knee flexors of the non-dominant leg, and with the conventional ratios of both the dominant and non-dominant leg (Table 7). Significant (*p* < 0.05) correlations were also observed between the SJ-A jump height and the relative torque of the knee extensors in the dominant leg and the knee flexors of the non-dominant leg.

As in the Men U18 group, the relative torque of the knee extensors of the non-dominant leg was significantly (*p* < 0.05) correlated with the jump height in all vertical jump tests. In addition, the angle to achieve peak knee extensor torque of the dominant leg was significantly (*p* < 0.05) correlated with the pre-stretch gain and the arm swing augmentation ratio. Finally, a significant (*p* < 0.05) correlation was revealed between the symmetry angle for the inter-limb difference concerning the angle to achieve the peak knee extensor torque and the pre-stretch gain and the CMJ-A jump height.

The comparison of the correlation coefficients between the concentric isokinetic parameters at the angular velocity of 300°/s and the vertical jump test performance parameters revealed significant (*p* < 0.05) sex differences between the Men U18 group and Women U18 group for the following relationships:

- Pre-stretch gain and (a) relative torque of the knee extensors of the dominant leg, (b) relative torque of the knee flexors of the dominant leg, (c) relative torque of the knee flexors of the non-dominant leg, (d) the angle to achieve peak knee extensor torque of the dominant leg, (e) the conventional H/Q ratio in the dominant leg, (f) the conventional H/Q ratio in the non-dominant leg, and (g) the symmetry angle of the inter-limb difference for the angle to achieve peak knee extensor torque (results favoring Women U18 athletes);
- Relative torque of the knee extensors of the non-dominant leg and (a) jump height of CMJ-A, and (b) jump height of CMJ-F (results favoring Men U18 athletes);
- Symmetry angle of the inter-limb difference for the angle to achieve peak knee extensor torque and jump height of CMJ-A (results favoring Women U18 athletes);
- Relative torque of the knee flexors of the non-dominant leg and jump height of SJ-A (results favoring Women U18 athletes).

## 4. Discussion

The results of this research indicated that top adolescent Men U18 track and field athletes had larger vertical jump performance, increased values in the kinetic parameters of the vertical jump and knee extensor torque values, and higher knee flexor torque in the non-dominant leg compared to the Women U18 athletes. Except for the force-output in the SJ-A, no sex differences in the magnitude of the relationship between vertical jump kinetics and jump height were observed. Significant sex differences were observed in the relationship between the isokinetic parameters and the vertical jump performance metrics: for the isokinetic tests at 60°/s, Women U18 athletes were found to rely more on the knee flexor torque than Men U18 athletes. In addition, for the isokinetic tests at 300°/s, Women U18 athletes exhibited stronger negative relationships between selected isokinetic metrics and the pre-stretch gain in the vertical jump tests compared to Men U18 athletes.

In the SJ-A, a significant sex difference was revealed regarding the relationship between jump height and relative force. The SJ-A is suggested to evaluate the concentric muscle strength capability [2] and the performance in this jump test was found to be largely dependent on the muscular strength of the leg extensor muscles [60]. This reliance on force to optimize SJ-A jump height was highlighted in a previous study, where young female track and field athletes were found to perform the SJ-A test with a distinct force-dependent pattern [10]. Due to the sex differences in power production capability as a result of the hormonal profile [61], women seemed to rely on the force output to achieve an optimum jump height in the SJ-A vertical jump test. On the other hand, no such difference was observed for the countermovement jumps. This could be attributed to the fact that, compared to SJ-A, a larger number of complex mechanisms with considerable variable combinations of their interaction are evident in the countermovement rather than the squat jump [62]. Nevertheless, the larger values for jump height and the kinetic parameters in the CMJ-A and CMJ-F in Men U18 than Women U18 group are in line with past findings of research examining sex differences in vertical jump tests [51,63].

The examined Men U18 track and field athletes had larger knee extensor torque for both the dominant and non-dominant leg than the Women U18 athletes. This is in agreement with past findings [38,39]. Again, the sex differences concerning the ability to apply higher muscular strength and power that is higher in men during maturity [42], which is related to more efficient force–time characteristics [64], can explain this advanced outcome in the examined Men U18 athletes [65]. This trend was revealed for both the isokinetic tests, with the lower average torque values observed in the isokinetic test at 300°/s compared to the respective values obtained in the isokinetic test at 60°/s. This observation is in agreement with past observations [66]. However, the sex difference was not evident for the knee flexor relative torque. The hamstrings are active and under considerable strain in a variety of athletics techniques, i.e., sprinting [67,68], long jumps [69,70], and throws [71,72].

It is considered that loading in the hurdle, the jumping, and the throwing disciplines is asymmetric in nature and has an impact on the tissues [73], a fact that is related to an increased risk of asymmetry and injury [74]. This inter-limb difference in the loading, as well as the demands in terms of physical capabilities and the constraints imposed by the sporting technique, is suggested to have an effect on the isokinetic metrics [66]. The sex difference in the symmetry angle for the inter-limb difference in the knee flexor relative torque can also be attributed to the fact that loading-imposed movement is greater in males than in females [75], and, in the case of sport technique, physical condition rather than coordination is the factor that results in sex differences in adolescent track and field athletes [76]. Gender differences in asymmetry exist in track and field, i.e., the step kinematics of the approach of the pole vault [77]. This difference should be further examined in the future, since past research provided evidence that concentric torque did not vary with leg dominance [78], no asymmetries were observed for track and field jumpers in strength and power tests [32], and the asymmetries in key biomechanical parameters of track and field techniques were not related to performance [79,80,81].

The results for the conventional H/Q ratio were in reasonable agreement with past findings [36,38], but considerably lower compared to the reported results for the adult members of the National Track and Field team [82]. No sex differences were observed, confirming past findings [38]. The importance of monitoring the H/Q ratio for evaluating performance and the risk of injury on a regular basis is emphasized in the literature [52].

The examined Women U18 athletes had significantly lower knee flexion torque compared to Men U18 athletes for the dominant leg in the 300°/s isokinetic test, confirming past findings [83]. This might be due to the fact that hamstring strength is maximized in female athletes by the age of 11 years, years earlier than male athletes [84]. In addition, no sex differences were observed concerning the angle to achieve peak torque. However, during the upward phase of the CMJ, males were found to present higher EMG activity than females in the knee extensor muscles, and higher co-activation levels were observed in females [85]. This higher co-activation ratio was attributed to increased limb stiffness [86]. Future studies should also examine the role of limb stiffness in the relationship between the vertical jump and the isokinetic parameters.

The relationship between the vertical jump and isokinetic parameters showed that torque values and jump height are interrelated [87]. In the case of the squat jump, isokinetic torque is suggested to be a fair predictor of the jump kinetic parameters [88]. In the present study, the performance in the vertical jump tests of the Women U18 group was found to be related to knee flexor relative torque during the 60°/s isokinetic test with significantly higher correlation coefficients than those observed in the examined Men U18 group. In addition, the pre-stretch gain in the Women U18 group had significantly higher negative correlation coefficients with the isokinetic parameters at the 300°/s angular velocity than the examined Men U18 athletes. Combined with the finding that no sex differences were revealed for the concentric knee flexor relative torque, further research is required to pinpoint the mechanisms that led the examined adolescent Women U18 athletes to present these differences.

The findings of this study should be generalized with caution, since there are some limitations. At first, the examination of athletes with uneven sample size between Men and Women U18 athletes from different disciplines could lead to the masking of unique associations among the outcomes of the experimental tests and the specific demands of the sport technique in each discipline. Future research should be conducted with adequate sample sizes taking into consideration the athletics discipline. In addition, no specific instruction was given for the knee flexion in the countermovement jump tests. This fact could have provided further information concerning the angle specificity between the experimental tests and the sport technique. Future research could be tailored to reveal the association between jumping biomechanics and isokinetic metrics based on the event and/or individual angle-specific profile.

## 5. Conclusions

Vertical jump and isokinetic tests are commonly used for the assessment of crucial parameters that are essential for high-level performance in track and field. The findings of this study suggest that Women U18 adolescent track and field athletes presented a stronger association of the knee flexor relative torque with vertical jump performance compared to Men U18 athletes. In addition, there was a sex difference related to the magnitude of the relationship between the conventional H/Q ratio and the pre-stretch gain. Therefore, it is proposed that adolescent track and field athletes should be regularly tested to monitor possible torque deficits of the muscles acting on the knee. Besides monitoring muscle imbalances to prevent injury, isokinetic metrics can also provide information for optimizing performance in explosive movements, i.e., vertical jumps, as knee torque is related to vertical jump performance parameters. However, coaches are suggested to implement a different approach in training based on the sex differences revealed for the relationship of jumping performance with isokinetic metrics, as jumping performance in Women U18 athletes was found to be more related to knee flexor torque than Men U18 athletes.

## Figures and Tables

**Table 1:** Results for the squat jump with arms akimbo, presented as mean, (standard deviation), [correlation coefficient with squat jump height], and the comparison between Men U18 and Women U18 track and field athletes.

Parameter	Men U18 (*n* = 21)	Women U18 (*n* = 14)	*t*	*p*	*g*	*g* [95% Confidence Interval]	
Jump height (m)	0.340 (0.046)	0.240 (0.035) *	6.235	<0.001	2.38	1.46	3.19
Impulse time (ms)	373.0 (39.5) [0.162]	403.0 (55.6) [−0.249]	1.691	0.103	0.64	0.06	1.32
Relative vGRF (N/kg)	2.69 (0.24) [0.201]	2.39 (0.20) * [0.730] ^†^*	3.565	0.001	1.33	0.56	2.04
Time to achieve peak vGRF (ms)	213.4 (55.6) [0.201]	262.8 (64.7) * [−0.315]	2.165	0.040	0.83	0.11	1.52
Rate of force development (kN/kg)	14.4 (5.7) [0.196]	8.5 (3.4) * [0.130]	3.166	0.004	1.20	0.44	1.90
Relative power (W/kg)	37.97 (5.30) **[0.828]** ^†^	28.37 (4.88) * [0.864]	4.904	<0.001	1.87	1.03	2.63
Time to achieve peak power (ms)	268.1 (38.9) [0.221]	298.2 (52.3) [−0.235]	1.745	0.093	0.67	0.35	1.04
Vertical BCM displacement (m)	0.510 (0.051) **[0.628] ^†^**	0.446 (0.033) * [0.375]	3.790	<0.001	2.38	1.46	3.19

Values typed in bold indicate significant correlations and sex differences. *: significant sex difference (*p* < 0.05); ^†^: significant correlation (*p* < 0.05) with jump height; vGRF: vertical ground reaction force; BCM: body center of mass.

**Table 2:** Results for the countermovement jump with arms akimbo, presented as mean (standard deviation), [correlation coefficient with squat jump height], and the comparison between Men U18 and Women U18 track and field athletes.

Parameter	Men U18 (*n* = 21)	Women U18 (*n* = 14)	*t*	*p*	*g*	*g* [95% Confidence Interval]	
Jump height (m)	0.372 (0.049)	0.256 (0.028) *	7.372	<0.001	2.76	1.77	3.62
Impulse time (ms)	567.0 (70.6) [0.056]	568.3 (75.1) [−0.532]	0.048	0.962	0.02	−0.69	0.66
Relative vGRF (N/kg)	2.85 (0.26) [0.377]	2.52 (0.19) * [0.493]	3.677	0.001	1.40	0.62	2.12
Time to achieve peak vGRF (ms)	305.8 (71.2) [0.207]	291.6 (49.5) [−0.349]	0.590	0.560	0.22	−0.46	0.90
Rate of force development (kN/kg)	15.1 (5.7) [0.184]	10.1 (3.5) * [0.273]	2.670	0.013	1.01	0.27	1.70
Relative power (W/kg)	35.77 (6.09) **[0.789] ^†^**	26.30 (3.91) * **[0.784] ^†^**	4.697	<0.001	1.77	0.94	2.52
Time to achieve peak power (ms)	458.1 (70.6) [0.064]	464.6 (74.0) [−0.507]	0.236	0.815	0.09	−0.76	0.59
BCM lowering (m)	−0.347 (0.046) [−0.321]	−0.291 (0.046) * [−0.160]	3.155	0.004	1.22	0.46	1.92
Vertical BCM displacement (m)	0.560 (0.063) [0.476]	0.483 (0.034) * [−0.182]	4.185	<0.001	1.44	0.65	2.16
Pre-stretch gain (%)	9.90 (10.41) [0.319]	6.82 (6.32) [−0.500]	0.908	0.340	0.35	−0.34	1.02

Values typed in bold indicate significant correlations and sex differences. *: significant sex difference (*p* < 0.05); ^†^: significant correlation (*p* < 0.05) with jump height; vGRF: vertical ground reaction force; BCM: body center of mass.

**Table 3:** Results for the countermovement jump with free arm swing, presented as mean (standard deviation), [correlation coefficient with squat jump height], and the comparison between Men U18 and Women U18 track and field athletes.

Parameter	Men U18 (*n* = 21)	Women U18 (*n* = 14)	*t*	*p*	*g*	*g* [95% Confidence Interval]	
Jump height (m)	0.452 (0.060)	0.300 (0.036) *	8.354	<0.001	2.95	1.98	3.92
Impulse time (ms)	613.8 (89.0) [−0.072]	604.5 (113.5) [0.032]	0.241	0.812	0.09	−0.59	0.77
Relative vGRF (N/kg)	2.79 (0.22) **[0.555] ^†^**	2.55 (0.21) * [0.196]	2.995	0.006	1.09	0.36	1.81
Time to achieve peak vGRF (ms)	470.8 (117.7) [0.005]	430.2 (138.0) [−0.063]	0.839	0.409	0.31	−0.37	0.99
Rate of force development (kN/kg)	12.2 (4.8) [−0.048]	10.0 (4.6) [−0.144]	1.170	0.253	0.46	−0.23	1.14
Relative power (W/kg)	47.43 (6.41) **[0.866] ^†^**	33.14 (3.36) * **[0.679] ^†^**	6.253	<0.001	2.58	1.67	3.48
Time to achieve peak power (ms)	506.0 (90.6) [−0.057]	500.1 (117.0) [0.052]	0.150	0.882	0.06	−0.62	0.73
BCM lowering (m)	−0.348 (0.064) [−0.324]	−0.314 (0.061) [0.356]	1.428	0.165	0.53	0.16	1.22
Vertical BCM displacement (m)	0.618 (0.057) [0.474]	0.503 (0.049) * [0.218]	5.569	<0.001	2.08	1.25	2.91
Arm swing augmentation ratio (%)	21.69 (6.64) [0.158]	17.44 (7.56) [−0.383]	1.579	0.126	0.60	−0.09	1.29

Values typed in bold indicate significant correlations and sex differences. *: significant sex difference (*p* < 0.05); ^†^: significant correlation (*p* < 0.05) with jump height; vGRF: vertical ground reaction force; BCM: body center of mass.

**Table 4:** Results for the concentric isokinetic tests at the angular velocity of 60°/s presented as mean (standard deviation) and the comparison between Men U18 and Women U18 track and field athletes.

Parameter	Men U18 (*n* = 21)	Women U18 (*n* = 14)	*t*	*p*	*g*	*g* [95% Confidence Interval]	
Dominant leg knee extensor relative torque (% body mass)	327.5 (43.0)	297.0 (28.6) *	2.168	0.038	0.78	0.08	1.49
Angle to achieve peak knee extensor torque—dominant leg (°)	71.6 (7.2)	74.3 (4.7)	1.170	0.251	0.42	0.27	1.10
Non-dominant leg knee extensor relative torque (% body mass)	333.2 (45.4)	281.3 (34.7) *	3.384	0.002	1.22	0.49	1.96
Angle to achieve peak knee extensor torque—non-dominant leg (°)	74.7 (7.7)	70.9 (6.8)	0.399	0.693	0.50	−0.18	1.19
Dominant knee flexor relative torque (% body mass)	216.1 (39.3)	185.6 (30.5) *	2.279	0.030	0.83	0.12	1.53
Angle to achieve peak knee flexor torque—dominant leg (°)	35.2 (9.5)	42.7 (11.5)	1.979	0.057	−0.71	−1.41	−0.01
Non-dominant knee flexor relative torque (% body mass)	191.6 (34.9)	167.2 (29.7)	2.010	0.054	0.72	0.03	1.42
Angle to achieve peak knee flexor torque—non-dominant leg (°)	38.6 (11.8)	39.5 (7.2)	0.230	0.820	−0.09	−0.76	0.59
Conventional H/Q ratio—dominant leg (%)	65.4 (11.3)	61.2 (7.7)	1.111	0.276	0.41	−0.27	1.09
Conventional H/Q ratio—non dominant leg (%)	58.5 (9.6)	60.6 (8.6)	0.610	0.547	−0.22	−0.90	0.46
Symmetry angle—knee extensor relative torque (%)	2.27 (2.05)	2.95 (1.73)	0.957	0.346	−0.34	−1.03	0.34
Symmetry angle—knee flexor relative torque (%)	3.80 (3.16)	3.36 (2.21)	0.418	0.679	0.15	−0.52	0.83
Symmetry angle—angle to achieve peak knee extensor torque (%)	2.23 (1.98)	2.82 (2.18)	0.775	0.444	−0.28	−0.96	0.40
Symmetry angle—angle to achieve peak knee flexor torque (%)	6.89 (5.44)	5.65 (4.4)	0.666	0.511	0.24	−0.44	0.92

Values typed in bold indicate significant correlations and sex differences. *: significant sex difference (*p* < 0.05).

**Table 5:** Correlation coefficients *r* for the relationship between the concentric isokinetic parameters at the angular velocity of 60°/s and the vertical jump tests performance parameters (jump height, elastic utilization ratio, and arm swing augmentation ratio) and the comparison between Men U18 and Women U18 track and field athletes.

Parameter	Men U18 (*n* = 21)					Women U18 (*n* = 14)				
	SJ-A	CMJ-A	CMJ-F	PSG	ASAR	SJ-A	CMJ-A	CMJ-F	PSG	ASAR
Dominant leg knee extensor relative torque	0.140	0.313	0.263	0.262	−0.168	**0.637 ^†^**	0.618	**0.663 ^†^**	−0.526	0.085
Angle to achieve peak knee extensor torque—dominant leg	0.152	0.077	0.117	−0.111	0.090	−0.380	−0.539	−0.524	0.079	−0.001
Non-dominant leg knee extensor relative torque	0.281	0.337	0.362	0.057	0.018	0.609	0.630	0.743 ^†^	−0.434	0.085
Angle to achieve peak knee extensor torque—non-dominant leg	−0.002	0.054	−0.021	0.095	−0.197	0.092	−0.087	0.154	−0.333	0.378
Dominant knee flexor relative torque	−0.195	−0.149	−0.063	0.017	0.252	0.919 ^†^*	0.781 ^†^*	0.889 ^†^*	−0.806 ^†^*	0.215
Angle to achieve peak knee flexor torque—dominant leg	−0.404	−0.227	−0.271	0.307	−0.144	−0.418	−0.595	−0.478	0.072	0.143
Non-dominant knee flexor relative torque	−0.160	−0.163	−0.090	−0.034	0.196	0.894 ^†^*	0.781 ^†^*	0.889 ^†^*	0.699 ^†^*	0.225
Angle to achieve peak knee flexor torque—non-dominant leg	**−0.591 ^†^**	−0.308	−0.494	0.460	−0.455	0.303 *	0.110	0.263	−0.465	0.234
Conventional H/Q ratio—dominant leg	−0.369	−0.489	−0.369	−0.207	0.358	0.674 ^†^*	0.514	0.599	−0.614	0.183
Conventional H/Q ratio—non-dominant leg	−0.296	−0.314	−0.264	−0.050	0.172	0.508	0.362	0.381	−0.435	0.061
Symmetry angle—knee extensor relative torque	−0.066	0.214	0.102	0.440	−0.299	−0.029	−0.117	−0.092	−0.094	0.006
Symmetry angle—knee flexor relative torque	−0.020	0.029	0.062	0.041	0.117	−0.220	−0.230	−0.253	0.009	−0.065
Symmetry angle—angle to achieve peak knee extensor torque	−0.086	0.040	0.005	0.215	−0.119	−0.059	0.072	−0.194	0.399	−0.431
Symmetry angle—angle to achieve peak knee flexor torque	0.085	−0.005	−0.003	−0.110	0.003	−0.551	−0.642 ^†^*	−0.503	0.264	0.185

Values typed in bold indicate significant correlations and sex differences. ^†^: significant correlation (*p* < 0.05), *: significant sex difference (*p* < 0.05); SJ-A: jump height at the squat jump with arms akimbo vertical jump test; CMJ-A: jump height at the countermovement jump with arms akimbo vertical jump test; CMJ-F: jump height at the countermovement jump with free arm swing test; PSG: pre-stretch gain; ASAR: arm swing augmentation ratio.

**Table 6:** Results for the concentric isokinetic tests at the angular velocity of 300°/s presented as mean (standard deviation) and the comparison between Men U18 and Women U18 track and field athletes.

Parameter	Men U18 (*n* = 21)	Women U18 (*n* = 14)	*t*	*p*	*g*	*g* [95% Confidence Interval]	
Dominant leg knee extensor relative torque (% body mass)	175.9 (21.5)	148.6 (12.4) *	3.834	<0.001	1.45	0.69	2.20
Angle to achieve peak knee extensor torque—dominant leg (°)	74.8 (21.7)	88.7 (26.5)	1.541	0.135	−0.57	−1.26	0.12
Non-dominant leg knee extensor relative torque (% body mass)	175.7 (29.8)	145.2 (20.4) *	2.986	0.006	1.12	0.40	1.85
Angle to achieve peak knee extensor torque—non-dominant leg (°)	73.9 (14.8)	82.0 (28.5)	1.012	0.321	−0.37	−1.05	0.31
Dominant knee flexor relative torque (% body mass)	123.4 (24.2)	100.1 (22.7) *	2.579	0.016	0.96	0.25	1.68
Angle to achieve peak knee flexor torque—dominant leg (°)	42.9 (21.6)	28.3 (22.1)	1.751	0.091	0.65	−0.04	1.35
Non-dominant knee flexor relative torque (% body mass)	108.8 (31.3)	99.1 (24.6)	0.874	0.390	0.33	−0.35	1.01
Angle to achieve peak knee flexor torque—non-dominant leg (°)	39.3 (10.8)	31.0 (22.8)	1.335	0.193	0.49	−0.20	1.17
Conventional H/Q ratio—dominant leg (%)	69.0 (9.2)	67.0 (11.4)	0.517	0.609	0.19	−0.48	0.87
Conventional H/Q ratio—non-dominant leg (%)	62.7 (12.3)	67.9 (11.2)	1.141	0.254	−0.43	−1.11	0.26
Symmetry angle—knee extensor relative torque (%)	2.97 (2.12)	2.46 (2.04)	0.640	0.527	0.24	−0.44	0.92
Symmetry angle—knee flexor relative torque (%)	6.57 (5.14)	3.05 (2.12) *	2.566	0.017	0.82	0.11	1.52
Symmetry angle—angle to achieve peak knee extensor torque (%)	3.45 (4.56)	4.76 (5.01)	0.723	0.476	−0.27	−0.95	0.41
Symmetry angle—angle to achieve peak knee flexor torque (%)	8.63 (7.99)	18.05 (39.14)	0.753	0.470	−0.36	−1.04	0.32

Values typed in bold indicate significant differences. *: significant sex difference (*p* < 0.05).

**Table 7:** Correlation coefficients *r* for the relationship between the concentric isokinetic parameters at the angular velocity of 300°/s and the vertical jump test performance parameters (jump height, elastic utilization ratio, and arm swing augmentation ratio) and the comparison between Men U18 and Women U18 track and field athletes.

Parameter	Men U18 (*n* = 21)					Women U18 (*n* = 14)				
	SJ-A	CMJ-A	CMJ-F	PSG	ASAR	SJ-A	CMJ-A	CMJ-F	PSG	ASAR
Dominant leg knee extensor relative torque	0.523	0.430	0.455	−0.126	0.067	**0.764 ^†^**	0.623	0.627	−0.753 ^†^*	0.058
Angle to achieve peak knee extensor torque—dominant leg	0.175	−0.315	−0.095	**−0.701 ^†^**	**0.637 ^†^**	−0.114	−0.304	−0.125	−0.188 *	0.277
Non-dominant leg knee extensor relative torque	**0.752 ^†^**	**0.919 ^†^**	**0.879 ^†^**	0.210	−0.165	0.597	0.459 *	0.598 *	−0.579	0.257
Angle to achieve peak knee extensor torque—non-dominant leg	0.485	0.271	0.390	−0.311	0.274	−0.155	−0.407	−0.195	−0.266	0.311
Dominant knee flexor relative torque	0.364	0.522	0.525	0.216	−0.044	0.621	0.335	0.602	−0.811 ^†^*	0.429
Angle to achieve peak knee flexor torque—dominant leg	−0.406	−0.267	−0.319	0.175	−0.048	−0.236	−0.078	−0.080	0.353	0.007
Non-dominant knee flexor relative torque	0.007	0.273	0.126	0.333	−0.349	0.683 ^†^*	0.439	0.589	−0.789 ^†^*	0.251
Angle to achieve peak knee flexor torque—non-dominant leg	−0.327	−0.287	−0.306	0.076	−0.021	−0.380	−0.199	−0.240	0.521	−0.075
Conventional H/Q ratio—dominant leg	−0.257	0.060	0.026	0.424	−0.123	0.476	0.173	0.500	**−0.720 ^†^**	0.508
Conventional H/Q ratio—non dominant leg	−0.373	−0.304	−0.290	0.076	0.061	0.488	0.262	0.361	−0.642 ^†^*	0.146
Symmetry angle—knee extensor relative torque	−0.207	−0.430	−0.308	−0.348	0.362	−0.046	−0.117	−0.205	−0.067	−0.177
Symmetry angle—knee flexor relative torque	0.479	0.403	0.514	−0.107	0.218	0.087	−0.010	−0.131	−0.167	−0.177
Symmetry angle—angle to achieve peak knee extensor torque	−0.134	**−0.567 ^†^**	−0.428	−0.619 ^†^	0.449	0.146	0.192 *	0.279	−0.010 *	0.143
Symmetry angle—angle to achieve peak knee flexor torque	−0.020	−0.102	0.046	−0.159	0.150	0.309	0.155	0.197	−0.415	0.071

Values typed in bold indicate significant correlations and sex differences. ^†^: significant correlation (*p* < 0.05), *: significant sex difference (*p* < 0.05); SJ-A: jump height at the squat jump with arms akimbo vertical jump test; CMJ-A: jump height at the countermovement jump with arms akimbo vertical jump test; CMJ-F: jump height at the countermovement jump with free arm swing test; PSG: pre-stretch gain; ASAR: arm swing augmentation ratio.

## Data Availability

The data presented in this study are available upon reasonable request from the corresponding author due to ethical reasons.
